# Edoxaban Monotherapy in Nonvalvular Atrial Fibrillation Patients with Coronary Artery Disease

**DOI:** 10.1155/2022/5905022

**Published:** 2022-12-17

**Authors:** Daisuke Fukamachi, Yasuo Okumura, Naoya Matsumoto, Eizo Tachibana, Koji Oiwa, Makoto Ichikawa, Hironori Haruta, Kazumiki Nomoto, Ken Arima, Atsushi Hirayama

**Affiliations:** ^1^Division of Cardiology, Nihon University Itabashi Hospital, Tokyo, Japan; ^2^Department of Cardiology, Nihon University Hospital, Tokyo, Japan; ^3^Kawaguchi Municipal Medical Center, Saitama, Japan; ^4^Yokohama Central Hospital, Yokohama, Kanagawa, Japan; ^5^Sekishindo Hospital, Saitama, Japan; ^6^TMG Asaka Hospital, Saitama, Japan; ^7^Tokyo Rinkai Hospital, Tokyo, Japan; ^8^Kasukabe Municipal Hospital, Saitama, Japan; ^9^Osaka Police Hospital, Osaka, Japan

## Abstract

**Background:**

Current guidelines recommend an oral anticoagulant (OAC) monotherapy in patients with nonvalvular atrial fibrillation (NVAF) and stable coronary artery disease (CAD) 1 year postpercutaneous coronary intervention (PCI). It might be possible to shorten the time for de-escalation from a dual therapy to monotherapy, but data regarding de-escalation to an edoxaban monotherapy are lacking. This study aimed to assess the clinical safety of an edoxaban monotherapy in patients with NVAF and stable CAD.

**Methods:**

A multicenter, prospective, randomized, open-label, and parallel group study was established to investigate the safety of an edoxaban monotherapy in patients with NVAF and stable CAD including over 6 months postimplantation of a third-generation DES and 1 year postimplantation of other stents (PRAEDO AF study). Between March 2018 and June 2020, 147 patients from 8 institutions in Japan were randomized to receive either an edoxaban monotherapy (*n* = 74) or combination therapy (edoxaban plus clopidogrel, *n* = 73). The primary study endpoint was the composite incidence of major bleeding and clinically significant bleeding, defined according to the ISTH criteria.

**Results:**

Major or clinically significant bleeding occurred in 2 patients in the monotherapy group (1.67% per patient-year) and in 5 patients in the combination therapy group (4.28% per patient-year) (hazard ratio, 0.39; 95% confidence interval, 0.08–2.02). There was no incidence of a myocardial infarction, stent thrombosis, unstable angina requiring revascularization, ischemic stroke, systemic stroke, or hemorrhagic stroke in either of the groups.

**Conclusions:**

The edoxaban monotherapy was shown to have acceptable clinical safety in patients with NVAF and stable CAD. The study was registered with the Japan Registry of Clinical Trials (jRCTs031180119).

## 1. Introduction

Four recent randomized control trials (RCTs) involving patients with nonvalvular atrial fibrillation (NVAF) and coronary artery disease (CAD), the PIONEER PCI [1], RE-DUAL PCI [2], AUGUSTUS [3], and ENTRUST-AF PCI [4] trials have shown a consistent reduction in the risk of bleeding, occurring any time during the first year after percutaneous coronary intervention (PCI) in patients treated by a dual therapy with any of 4 direct-acting anticoagulants (DOACs) (dabigatran, rivaroxaban, apixaban, or edoxaban) and single antiplatelet therapy (SAPT), and that the risk of an ischemic event is equivalent between the dual therapy and triple therapy with a vitamin K antagonist (VKA) plus dual antiplatelet therapy (DAPT). Accordingly, the recent European, American, and Japanese guidelines for patients with AF undergoing PCI (5–7) typically recommend that a triple therapy needs to be de-escalated to a dual therapy within 1 month after stenting and that the dual therapy be maintained up to 1 year after the stenting [5–7]. Moreover, in patients at low ischemic risk as well as those at high risk for bleeding, it is reasonable to discontinue the SAPT 6 months after the PCI [5, 6]. Furthermore, DOACs are preferable to warfarin and other VKAs [5–7]. A registered observational study conducted in Denmark showed that in comparison to the use of a VKA alone, the combined use of a VKA and aspirin or thienopyridine increased the occurrence of bleeding complications by 1.50- to 1.84-fold among patients with AF and stable CAD [8]. The efficacy and safety of an oral anticoagulant (OAC) monotherapy were recently supported by data from 2 RCTs performed in Japan. The OAC ALONE trial [9] was a trial that mainly investigated a warfarin monotherapy as the counterpart to a combination therapy (warfarin/rivaroxaban plus SAPT). This randomized trial did not establish noninferiority of an OAC alone to a combined OAC and SAPT in patients with atrial fibrillation and chronic coronary syndrome beyond 1 year after stenting. The antithrombotic therapy for atrial fibrillation with stable coronary disease (AFIRE) study [10] showed that a rivaroxaban monotherapy was superior to a dual therapy in terms of the safety and had a noninferior efficacy in patients with NVAF and stable CAD. The recent European, American, and Japanese guidelines for patients with AF undergoing PCI generally recommend the administration of an OAC monotherapy for 1 year after the PCI in patients with NVAF and stable CAD, but data exist only for a VKA or rivaroxaban monotherapy [5–7]. Although edoxaban is widely used in Japan for patients with NVAF, data are lacking regarding the efficacy and safety of the edoxaban monotherapy in patients with NVAF and stable CAD.

Stent technology has advanced rapidly in recent years. Persistent polymer stents have been designed to solve the long-term safety (especially very late stent thromboses) and efficacy issues of the first-generation and second-generation drug-eluting stents (DESs). A third-generation DES was developed to promote early and rapid healing of the neointima, and the polymer was absorbed within 4 months [11]. Thus, shortening the time for a de-escalation from a dual therapy to an OAC monotherapy may become a preferred option, reducing the bleeding risk, and at the same time preventing stent thromboses. To clarify any unresolved issues, we conducted an RCT, which we refer to as the PRAEDO AF study (Prospective RAndomized study of safety outcomes treated with EDOxaban in patients with stable CAD and atrial fibrillation), to examine the efficacy and safety of an edoxaban monotherapy in patients with NVAF and stable CAD including over 6 months after the implantation of a third-generation DES and 1 year after the implantation of other stents.

## 2. Methods

### 2.1. Study Design and Patient Enrollment

The PRAEDO AF study was conducted in Japan as a multicenter, prospective, randomized, open-label, and parallel-group study between March 30, 2018 and June 30, 2021. The study was registered with the Japan Registry of Clinical Trials (jRCTs031180119), and the study design and study rationale were previously described [12]. In summary, 8 hospitals participated. Patients with NVAF and stable CAD including over 6 months after the implantation of a third-generation DES and 1 year after the implantation of other stents were enrolled between March 2018 and June 2020. All provided written informed consent for their participation and use of their anonymized data for the study purposes. All were followed up for at least 1 year. Inclusion criteria were as follows: (1) a clinical diagnosis of NVAF and stable CAD, (2) a CHADS2 score ≥1, and (3) age ≥20 years. In this study we defined stable CAD as follows: (1) patients must be stable for at least 6 months after the PCI. In addition, the patients must have had a third-generation stent implanted if they entered the study 6–12 months after the PCI, (2) stenotic lesions without ischemia requiring a PCI on coronary CT or coronary angiography, and (3) nonoccurrence of an acute coronary event for at least 6 months after coronary artery bypass grafting (CABG). Exclusion criteria were as follows: (1) a contraindication to the use of edoxaban, (2) a contraindication to the use of clopidogrel, (3) a scheduled revascularization, (4) an intended open surgery (other than gastrointestinal endoscopic biopsy), (5) an active tumor and uncontrolled hypertension (clinic systolic pressure ≥160 mmHg, and (6) any condition rendering the patient unsuitable for the study, as judged by the research director or research coordinator. The study was conducted in accordance with the Declaration of Helsinki and the Ethical Guidelines for Clinical Research issued by the Ministry of Health, Labour, and Welfare of Japan.

### 2.2. Randomization

The patients were randomly assigned, by means of an allocation table, to the use of edoxaban alone or edoxaban plus, an antiplatelet agent, at 1 : 1 ratio. The edoxaban was administered orally once daily at 60 mg, and it was then reduced to 30 mg once daily for patients with a creatinine clearance rate of 15–50 mL/min or body weight of <60 kg. The antiplatelet agent used in the edoxaban plus antiplatelet drug combination group was clopidogrel, a thienopyridine prodrug.

### 2.3. Data Collection

The following information was obtained from each patient's clinical record at the time of enrollment: date, when the edoxaban was started and the dosage; type of stent (first-generation DES (Cypher® or TAXUS®), second-generation DES (PROMUS®, XIENCE®, or Endeavor®), or third-generation DES (SYNERGY®, Orsiro®, or Ultimaster®)); clinical characteristics, including age and sex, body weight and height, systolic/diastolic blood pressure, heart rate, risk scores (CHADS2, CHA2DS2-VASc, and HAS-BLED scores), and type of AF (paroxysmal, persistent, or long-lasting AF); medications currently used; any comorbidity (hypertension, diabetes, stroke, other vascular disease, congestive heart failure, and/or peripheral artery disease); current habitual use of tobacco and/or alcohol; and medical history, including a previous coronary intervention or CABG, any previous surgery, any other intervention, or a previous malignancy. Patients generally underwent follow-up examinations at 1 month after enrollment and every 3 months thereafter. Blood samples were obtained at the time of enrollment, 6 months later, and at the final follow-up examination to determine the patient's blood count, liver function, renal function, and BNP or NT-proBNP level.

### 2.4. Management of Patient Information and Follow-Up Data

A website was created for the PRAEDO AF study and featured a web-based registration system by which the patient information was entered into a database. Each patient's clinical information and follow-up data were entered by the clinical physician or a clinical research coordinator into online forms and saved to the database. The data entry was checked by data managers at the central registry office. Information pertaining to each patient enrolled in the study was checked, through the central registry office, twice a year for up to 3 years and updated if necessary. For all enrollees, those in whom an event had occurred and those in whom an event had not occurred, the continuation or termination of antithrombic drugs, body weight, specific laboratory test results, and medications used were obtained routinely and recorded. If a patient was transferred to another hospital and discontinued or completed the antithrombic drugs during the follow-up period, the information was collected as long as possible until the follow-up period ended.

### 2.5. Study Endpoints

The primary study endpoint was the incidence of major bleeding and clinically significant bleeding events combined, defined according to the ISTH (International Society on Thrombosis and Haemostasis) criteria [13] for each of the 2 study groups. Clinically significant bleeding was defined as bleeding requiring treatment or tests, for example, a hospitalization or extension of the hospitalization period, laboratory tests, imaging, endoscopy, colonoscopy, cystoscopy, bronchoscopy, nasal packing, coil embolization, inotropic therapy, surgery, or interruption or discontinuation of anticoagulation, as advised by the patient's physician, or a change in a concomitant treatment, as advised by the patient's physician.

The secondary study endpoints were comprised of the following: (1) the composite of a myocardial infarction, stent thrombosis, and unstable angina pectoris requiring revascularization, (2) the composite of an ischemic stroke, systemic embolism, and cardiovascular death, (3) a myocardial infarction, (4) a stent thrombosis, (5) unstable angina requiring revascularization, (6) an ischemic stroke/systemic embolism, (7) minor bleeding, (8) cardiovascular death, and (9) noncardiovascular death.

### 2.6. Sample Size and Calculation Data

The study was designed to compare the safety of a treatment with edoxaban alone and that of a conventional treatment with edoxaban and antiplatelet drugs in terms of the incidences of major bleeding and clinically significant bleeding events. In the J-ROCKET study [14], which focused on the DOAC rivaroxaban, the combined incidence of major bleeding and clinically significant bleeding was 14% (major bleeding: 2.5%) with the use of rivaroxaban alone and 20.6% (major bleeding: 6.6%) with the use of antiplatelet drugs. A subanalysis of the ENGAGE AF-TIMI48 [15] trial showed a total bleeding event rate of 9.72% for an edoxaban monotherapy and 14.93% for edoxaban plus antiplatelet drugs. In addition, a previous study showed the risk of a major hemorrhagic event associated with the use of warfarin and clopidogrel in combination, rather than warfarin alone, to be increased, at a hazard ratio (HR) of 1.84 [8]. Patients with NVAF and stable CAD are considered to have a similar or greater risk of bleeding events, with the estimated combined incidence of major bleeding and clinically significant bleeding events being 13% for the edoxaban monotherapy group versus 30% for the edoxaban plus clopidogrel group. To achieve a power of 80%, sample sizes of 90 were needed for each group. Considering a drop-out rate of 10%, the target number of patients required for enrollment was set at 200 (100 per group). Nonetheless, because of a slow enrollment, the study was extended, but the steering committee decided to terminate the enrollment prematurely on June 30, 2020.

### 2.7. Statistical Analysis

Data are shown as the mean ± SD values, median (25^th^, 75^th^ percentiles), or number (percentage) of patients. Between-group differences among the continuous variables were analyzed by a Student's *t*-test and differences in the categorical variables were analyzed by a chi-square test or Fisher's exact text. All outcome analyses were performed on an intention-to-treat basis. A Cox proportional-hazards model was used to compare the outcomes between the 2 groups, with the results expressed as a hazard ratio (HR) and 95% confidence interval (CI). All statistical analyses were performed with SAS 9.4 for Windows (SAS Institute, Cary, NC).

## 3. Results

### 3.1. Study Population

**Figure 1:**
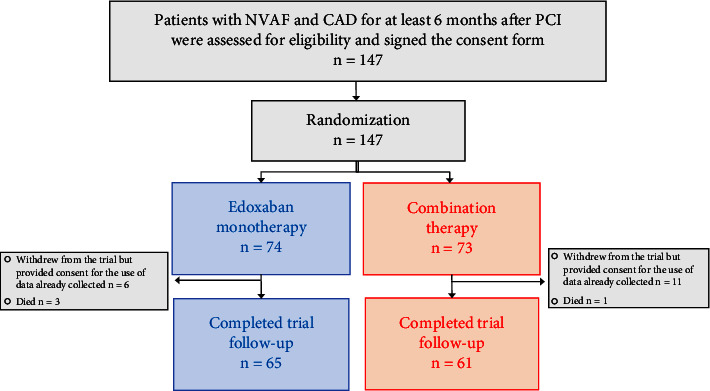
Flowchart showing the enrollment, randomization, and follow-up of the study patients. The patients were randomly assigned at a 1 : 1 ratio to receive a monotherapy with edoxaban or a combination therapy with edoxaban and clopidogrel.

One hundred forty-seven patients were enrolled in the study, with 74 assigned to the edoxaban monotherapy group and 73 to the combination therapy group (Figure 1). Sixty-five patients in the monotherapy group and 61 in the combination therapy group completed the final follow-up examination. The characteristics of the patients at the time of enrollment were similar between the 2 groups (Table 1). The mean age of the patients in both groups was 74 years. Eighty-seven percent of the patients in the edoxaban monotherapy group and 86% in the combination therapy group were men. Sixty-four (87%) patients in the monotherapy group and 60 (83%) in the combination therapy group underwent a previous PCI. The use of a P2Y12 inhibitor before enrollment was significantly less prevalent among the patients in the edoxaban monotherapy group than among those in the combination therapy group (46% vs. 66%, respectively; *p*=0.020). The time from the PCI to enrollment of the patients given a first-generation or second-generation DES was 1966 (1516, 3116) days for the monotherapy group and 1933 (1158, 2858) days for the combination therapy group (*p*=0.67), and that for the patients given a third-generation DES (SYNERGY, Orsiro, or Ultimaster) was 329 (206, 514) days and 310 (257, 514) days, respectively (*p*=0.77). In both groups, the median CHADS2 score was 2, median CHA2DS2-VASc score 4, and median HAS-BLED score 3.

### 3.2. Clinical Outcomes

**Figure 2:**
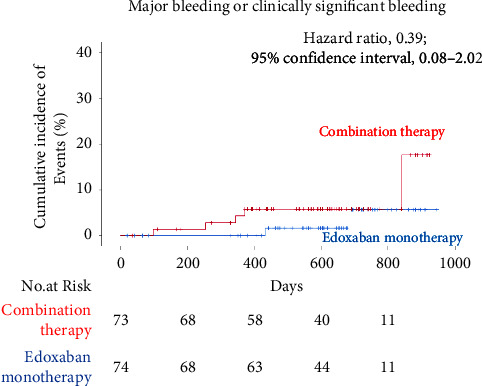
Kaplan–Meier curves for the cumulative incidence of the primary endpoint (occurrence of major bleeding or clinically significant bleeding) in the edoxaban monotherapy group and combination therapy group.

During the median follow-up period of 624 (range: 457–722) days, the primary endpoint, i.e., major or clinically significant bleeding, occurred in 2 patients (1.67% per patient-year) in the edoxaban monotherapy group and in 5 (4.28% per patient-year) in the combination therapy group (HR: 0.39; 95% CI: 0.08–2.02) (Table 2, Figure 2). Cardiovascular death, a secondary endpoint, occurred in 1 patient in the edoxaban monotherapy group. Two patients given edoxaban monotherapy and 1 given the combination therapy suffered a noncardiovascular death. There were no incidences of a myocardial infarction, stent thrombosis, unstable angina requiring revascularization, ischemic stroke, systemic stroke, or hemorrhagic stroke in either group. Minor bleeding occurred in 4 patients (3.35% per patient-year) in the monotherapy group and in 4 (3.43% per patient-year) in the combination therapy group (HR: 0.43; 95% CI: 0.13–1.41).

## 4. Discussion

The PRAEDO AF study described herein showed the feasibility of the application of an edoxaban monotherapy instead of edoxaban plus clopidogrel in patients with NVAF and stable CAD including over 6 months after the implantation of a third-generation DES and 1 year after the implantation of other stents. Our PRAEDO AF trial showed a lower incidence of the primary endpoint (i.e., occurrence of major or clinically significant bleeding) (at 1.67% per patient-year vs. 4.28% per patient-year) in the edoxaban monotherapy group than in the combination therapy group. In the AFIRE study, the incidence of major bleeding was found to be 1.62% per patient-year in the monotherapy group and 2.76% per patient-year in the combination therapy group, but our PRAEDO AF trial showed no major bleeding in either of the groups. Although rivaroxaban, apixaban, and edoxaban are all direct factor Xa inhibitors, different pharmacokinetics have been reported, with edoxaban having a lower bioavailability than rivaroxaban (45% vs. >80%) [16], and the lowest geometric mean area under the plasma concentration time curve of the 3 direct factor Xa inhibitors compared [17]. These pharmacokinetic properties of edoxaban may explain, at least partially, why numerous clinical studies have shown a lower incidence of major bleeding events in patients with NVAF given edoxaban than in those given rivaroxaban [18, 19]. The results of our moderately sized (underpowered) study, taken together with the reported data, may aid in clarifying the clinical potential (particularly in terms of safety) of an edoxaban monotherapy in patients with NVAF and stable CAD including over 6 months after the implantation of a third-generation DES and 1 year after the implantation of other stents.

The results of our trial lend clinical insight into the potential for shortening the time for de-escalation from a dual therapy to an edoxaban monotherapy in patients given a third-generation DES. Most DESs (47%) used in our study patients were third-generation DESs (SYNERGY systems). The median time from the SYNERGY implantation to the study enrollment was 313 days for the patients in the edoxaban monotherapy group versus 293 days for those in the combination therapy group. Importantly, this study revealed a similar absence of thrombotic events, i.e., of stent thromboses, myocardial infarctions, and unstable angina, between the edoxaban monotherapy group and combination therapy group. The target lesion revascularization 6 months after the implantation of a third-generation DES has been shown to be comparable to that following an implantation of a conventional second-generation DES, with no in-stent thromboses, myocardial infarctions, or cardiac death [20]. Third-generation DESs have yielded complete endothelialization within 28 days of the implantation in porcine coronary arteries [11]. Edoxaban has been shown in at least one animal study to exert antithrombotic effects [21] and in a human in-vitro study to exert anti-inflammatory effects [22]. In considering the response of the intima to third-generation DESs and the antiplatelet, anticoagulant, and anti-inflammatory activity of edoxaban, the results of our study pointed to the clinical acceptability of a shortened de-escalation from a dual therapy to an edoxaban monotherapy.

### 4.1. Study Limitations

First, the number of study patients was lower than planned because of slow entry and COVID-19 pandemic. Furthermore, the incidence of bleeding events was much lower than expected. Thus, our study was statistically underpowered to determine the true safety. Second, the open-label design may have led to a bias. Third, the edoxaban dosage differed between the edoxaban monotherapy and combination therapy regimens based on the renal function and body weight to guide lower doses of edoxaban. Furthermore, the pre-enrollment use of P2Y12 inhibitors was significantly lower in the edoxaban monotherapy group than in the combination therapy group, and this difference could have influenced the risk of bleeding events. However, no bleeding events occurred within 3 months after the start of clopidogrel, and thus, this effect, if any, was likely to have been small. Finally, the DES systems that were used varied among the study patients despite the similar prevalence of the various DESs used in the 2 groups.

## 5. Conclusions

Edoxaban monotherapy may have a clinically acceptable safety profile in NVAF patients with stable CAD including over 6 months after the implantation of a third-generation DES and 1 year after the implantation of other stents.

## Figures and Tables

**Table 1:** Characteristics of the patients on enrollment, per study group.

	Edoxaban monotherapy (*n* = 74)	Combination therapy (*n* = 73)	*P* value^*∗*^
Age, years	74 ± 7	74 ± 9	0.91
Male sex	64 (87)	63 (86)	0.97
BMI (m^2^/kg)	25 ± 4.9	24 ± 4.1	0.28
Weight (kg)	66 ± 15	65 ± 14	0.59
Height (cm)	162 ± 7.2	163 ± 7.4	0.62
Comorbidities			
Current smoking	8 (11)	9 (12)	0.89
Hypertension	65 (88)	63 (86)	0.78
Diabetes mellitus	29 (39)	34 (47)	0.37
Hepatic function abnormal	3 (4)	1 (1)	0.62
Medical history			
Previous PCI	64 (87)	60 (82)	0.47
Previous CABG	5 (7)	8 (11)	0.40
Myocardial infarction, peripheral artery disease, and aortic plaque	60 (81)	51 (70)	0.11
Previous stroke, transient ischemic attack, or systemic embolism	9 (12)	12 (16)	0.46
Valvular disease	3 (4.1)	4 (5.5)	0.88
Cancer	2 (2.7)	3 (4.1)	0.68
Past bleeding complications	10 (14)	8 (11)	0.90
Type of stent			
Drug eluting	45 (61)	47 (64)	0.56
Bare metal	9 (12)	4 (6)	
POBA	8 (11)	9 (12)	
Only medication	12 (16)	13 (18)	
DES system			
SYNERGY	18 (45)	25 (53)	0.79
Orsiro	1 (2)	1 (2)	
Ultimaster	7 (16)	2 (4)	
Xience	5 (11)	6 (13)	
Promus	6 (13)	5 (11)	
Resolute integrity	2 (4)	3 (6)	
Endeavor	1 (2)	0 (0)	
TAXUS	1 (2)	1 (2)	
Cypher	2 (4)	2 (4)	
Unknown	2 (4)	2 (4)	
Time from PCI to enrollment (d)			
SYNERGY	313 (212, 473)	293 (243, 504)	0.99
Orsiro	201	324	
Ultimaster	393 (206, 779)	1119	0.05
Xience	1966 (422, 2799)	979 (257, 2043)	0.43
Promus	1926 (1619, 2282)	2407 (1910, 3358)	0.15
Resolute integrity	1536	1505	0.75
Endeavor	2444		
TAXUS	4379	2615	
Cypher	4776	4750	0.92
1^st^ or 2^nd^ generation DES	1966 (1516, 3116)	1933 (1158, 2858)	0.67
3^rd^ generation DES (SYNERGY, orsiro, ultimaster)	329 (206, 514)	310 (257, 514)	0.77
Bare metal stent	4666 (3836, 6492)	2927 (2304, 3273)	0.06
POBA	1485 (658, 4275)	974 (397, 2095)	0.63
Type of AF			
Paroxysmal	13 (18)	14 (19)	0.91
Persistent	48 (65)	48 (65)	
Permanent	13 (18)	11 (16)	
Previous intervention other than PCI			
Catheter ablation	7 (10)	9 (12)	0.69
Pacemaker implantation	3 (4)	3 (4)	
Implantable cardioverter defibrillator	4 (5)	1 (1)	
Cardiac resynchronization therapy	1 (1)	2 (3)	
Prior antithrombotic drug therapy			
Anticoagulants used			
Warfarin	2 (3)	1 (1)	0.51
Dabigatran	5 (7)	1 (1)	0.11
Rivaroxaban	7 (10)	7 (10)	0.60
Apixaban	4 (6)	6 (8)	0.36
Edoxaban	54 (73)	55 (75)	0.74
Antiplatelets used			
Aspirin	29 (39)	26 (36)	0.65
P2Y12	34 (46)	48 (66)	0.020
Cilostazol	3 (3)	1 (1)	1.0
None	13 (18)	5 (7)	0.08
Initial dose of edoxaban (mg)			
60 mg	36 (49)	29 (40)	0.36
30 mg	38 (51)	43 (59)	
15 mg	0	1 (1)	
Use of proton pump inhibitors	59 (80)	64 (88)	0.19
CHADS2 score	2 (median)	2 (median)	
0	0 (0)	0 (0)	0.25
1	14 (19)	9 (12)	
2	33 (45)	27 (37)	
3	16 (22)	23 (32)	
4	5 (7)	11 (15)	
5	5 (7)	3 (4)	
6	1 (1)	0 (0)	
CHA2DS2 -VASc score	4 (median)	4 (median)	
0	0	0 (0)	0.26
1	1 (1)	0 (0)	
2	4 (5)	7 (10)	
3	13 (18)	13 (18)	
4	31 (42)	21 (29)	
5	14 (19)	16 (22)	
6	5 (7)	13 (18)	
7	5 (7)	3 (4)	
8	1 (1)	0 (0)	
HAS-BLED score	3 (median)	3 (median)	
0	0 (0)	0 (0)	0.68
1	4 (5)	2 (3)	
2	17 (23)	16 (22)	
3	34 (46)	35 (48)	
4	16 (22)	18 (25)	
5	1 (1)	2 (3)	
6	2 (3)	0 (0)	
Laboratory test results			
Hb	13.6 ± 1.7	13.4 ± 1.9	0.53
Creatinine clearance	58.3 ± 14	56.8 ± 16	0.52
NTproBNP	235 (151, 513)	411 (140, 661)	0.20

Data are shown as the number (%) of patients, mean ± SD values, or median (25^th^, 75^th^ percentile) values. % = % of the total of 45 and 47 patients who underwent a DES implantation in the edoxaban monotherapy group and combination therapy group, respectively. AF, atrial fibrillation; BMI, body mass index; CABG, coronary artery bypass grafting; DES, drug eluting stent; Hb, hemoglobin; NT-pro BNP, N-terminal probrain natriuretic peptide; PCI, percutaneous coronary intervention; POBA, plain old balloon angioplasty. ^*∗*^Determined by the *t*-test, chi-square test, or fisher's exact test.

**Table 2:** Primary and secondary endpoints, per study group.

	Edoxaban monotherapy (*n* = 74)	Combination therapy (*n* = 73)	Hazard ratio (95% CI)	*P* value
Primary endpoint				
Major bleeding or clinically significant bleeding	2 (1.67) (major bleeding: 0)	5 (4.28) (major bleeding: 0)	0.39 (0.08–2.02)	0.26
Secondary endpoints				
Cardiovascular event (s)				
Myocardial infarction, stent thrombosis, and unstable angina requiring revascularization	0	0		
Ischemic or systemic stroke and cardiovascular death	1	0		
Myocardial infarction	0	0		
Stent thrombosis				
Unstable angina requiring revascularization	0	0		
Ischemic stroke and systemic embolism	0	0		
Minor bleeding	4 (3.35)	4 (3.43)	0.43 (0.13–1.41)	0.97
Death				
Cardiovascular	1	0		
Noncardiovascular	2 (1.66)	1 (0.86)	1.99 (0.18–21.9)	0.58

Values are the number of patients (% per patient-year). CI, confidence interval.

## Data Availability

The data used to support the findings of this study are available from the corresponding author upon request.
